# Supplementary material for: The Derived Components of Gnaphalium hypoleucum DC. Reduce Quorum Sensing of Chromobacterium violaceum
Source: Molecules. 2022 Jul 30;27(15):4881. doi: 10.3390/molecules27154881 (PMC9369693; doi:10.3390/molecules27154881)
Supplement: Supplementary file 1 [file molecules-27-04881-s001.zip › molecules-1799318-supplementary.pdf]

The derived components of *Gnaphalium hypoleucum* DC. reduce quorum sensing of *Chromobacterium violaceum*

Yu-Long Li[1,2] and Zi-Yong Chu[2], Gui-Min Liu[2], Sheng-qiang Yang[1#], Hong Zeng[1*#]
[1]School of Basic Medicine, Youjiang Medical University for Nationalities, Baise, 533000, Guangxi, PR China

[2]College of Life Science and technology, Key Laboratory of Protection and Utilization of Biological Resources in Tarim Basin of Xinjiang Production & Construction Corps, Tarim University, Alar, China

[*]**Corresponding author**:

Hong Zeng*[1]: School of Basic Medicine, Youjiang Medical University for Nationalities, Baise, 533000, Guangxi, PR China. zenghong0705@163.com

#：The two authors are same contribution

Abstract

Gnaphalium hypoleucum DC. was first recorded in the Chinese National Pharmacopoeia "Yi Plant Medicine". There is no detailed report on its main components' activity in suppressing quorum sensing activity (QS) of bacteria. Our study aimed to screen the main components in extracts of *G. hypoleucum* DC. to measure their effects on bacterial QS activity and to explore specific quorum sensing mechanisms that are affected by *G, hypoleucum* DC. extracts.

Crude extracts of *G. hypoleucum* DC. contained significant amounts of two compounds shown to inhibit bacterial QS activity: apigenin and luteolin. Apigenin and luteolin in crude extracts of *G. hypoleucum* DC. showed substantial inhibition of pigment formation, biofilm production, and motility in *C. violaceum* ATCC 1247 compared to the effects of other phytochemicals from *G.*

hypoleucum DC. Apigenin and luteolin exhibited a strong QS inhibitory effect on *C. violaceum* ATCC12472, interfering with the violacein pigment biosynthesis by downregulating the *vio* B, *vio* C, and *vio* D genes. In the presence of signal molecules, the QS effect is prevented and the selected compounds can still inhibit the production of the characteristic purple pigment in *C. violaceum*.

Based on qualitative and quantitative research using genomics and bioinformatics, apigenin and luteolin in crude extracts of *G. hypoleucum* DC can interfere with the generation of QS in *C. violaceum* by downregulating the *vio* B, *vio* C, and *vio* D genes. *G. hypoleucum* DC is used for the treatment of bacterial infections and provides new ideas and potential alternative uses for medicinal plants.

Keywords

Quorum sensing; *Gnaphalium hypoleucum* DC.; Molecular docking; *C. violaceum* ATCC 12472; Biofilm

Contents

Figure S1. Flavonoids extracted from *Gnaphalium hypoleucum* DC.

Figure S2. The HPLC of flavonoids extracted from *Gnaphalium hypoleucum* DC.

Figure S3. C_{10}-HLS, apigenin and luteolin to CviR activity pocket molecular docking prediction map.

Table S1. Information on primers used in the design of experiments

Table S2. Minimum inhibitory concentration of extracts and compounds from *G. hypoleucum* DC against *C. violaceum* ATCC12472

Table S3. Docking and binding energy data of each compound molecule to CviR

Table S4. Table S4 Detailed data for calculations in molecular docking

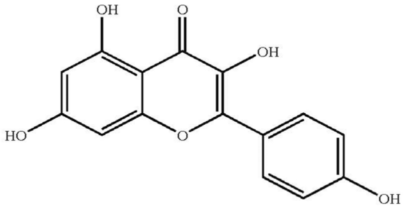

a

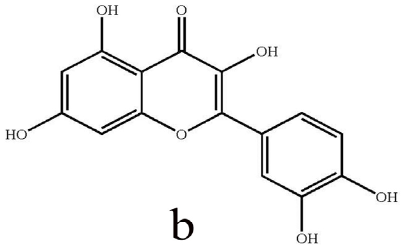

b

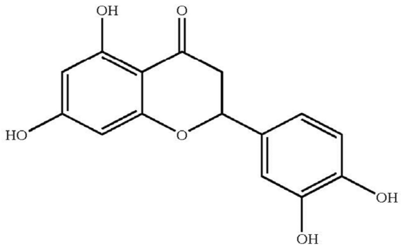

c

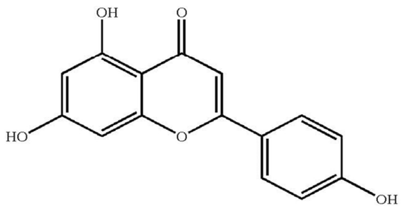

d

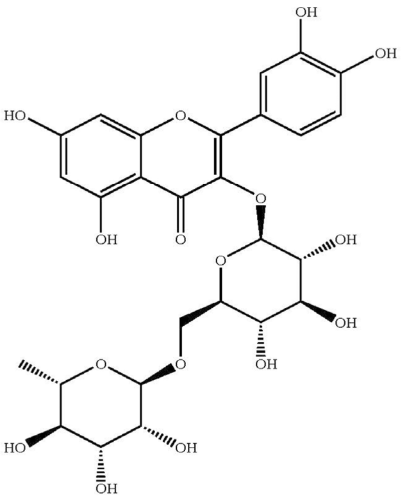

e

Figure S1. Flavonoids extracted from *Gnaphalium hypoleucum* DC.

Note： (a) kaempferol, (b) quercetin, (c) luteolin, (d) apigenin, (e) rutin.

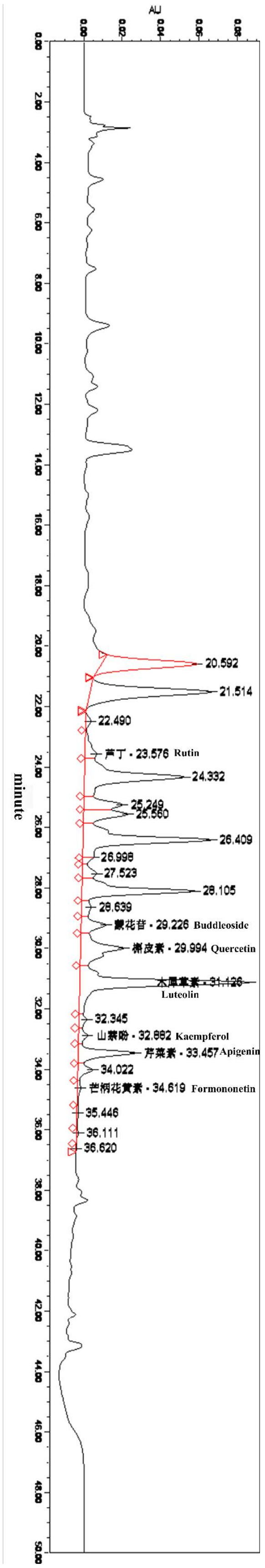

Figure S2. The HPLC of flavonoids extracted from *Gnaphalium hypoleucum* DC.

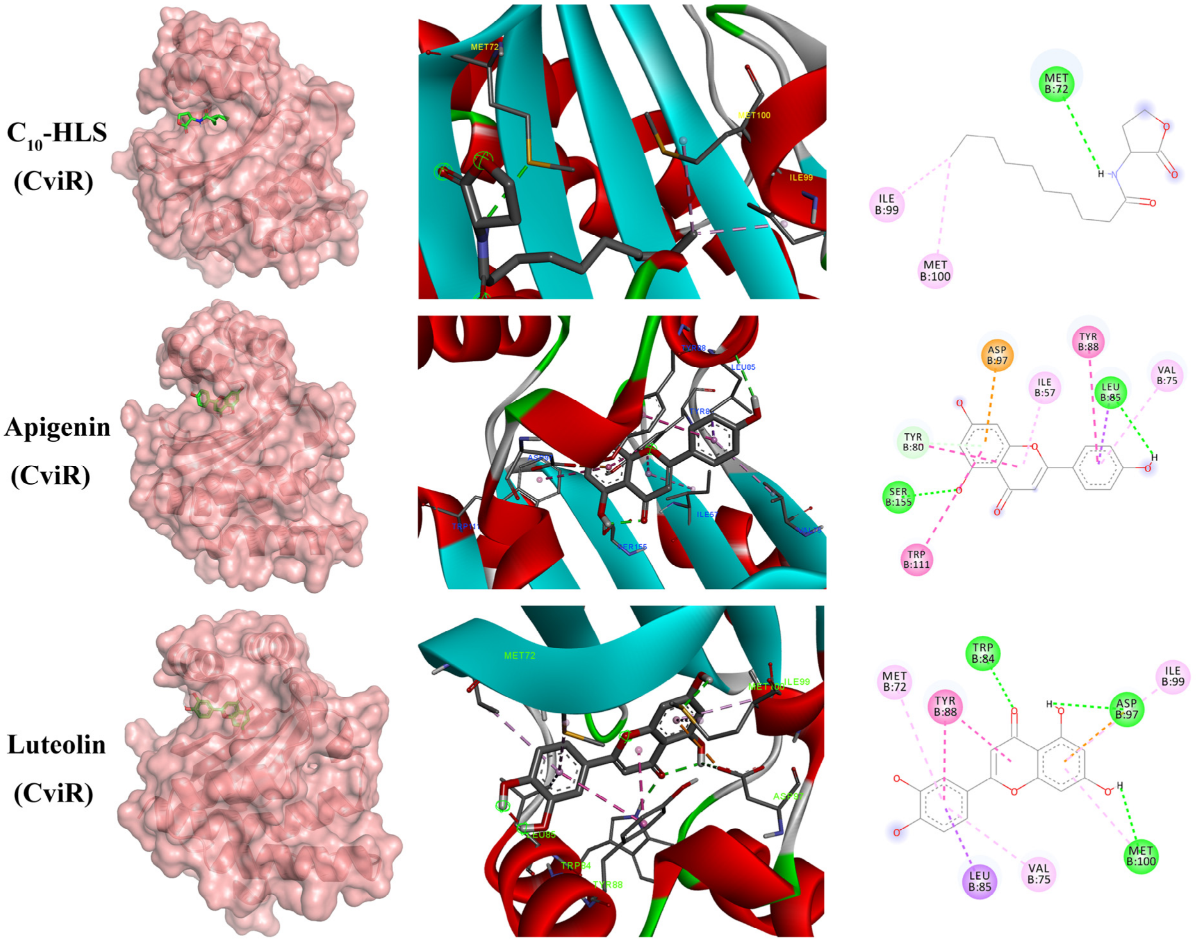

Figure S3. C10-HLS, apigenin and luteolin to CviR activity pocket molecular docking prediction map. The left side is the three-dimensional structure of the connection position, and the middle and right side is the label of the specific binding site.

Table S1. Information on primers used in the design of experiments

ID	primer name	sequence (5'to3')
16s	16s-F	GCGCAACCCTTGTCCTTAGTT
	16s-R	TGTCACCGGCAGTCTCCTTAG
vioA	*vioA*-2F	AAGAGCATGGCAAGGAATCC
	vioA-2R	CACTGGTTGGCGTCGTTAT
vioB	*vioB*-2F	TGGAACAGGAAGTGCGGATG
	vioB-2R	CCGGCAACAACCATTTCTCC
vioC	*vioC*-2F	GATCATATCGCCCTGCAAGC
	vioC-2R	CCGCTACTACTTCGAGCACA
vioD	*vioD*-2F	GTACTCGGACACGATGAGCAC
	vioD-2R	CACCTTGGCGACGTATTCGG

Table S2. Minimum inhibitory concentration of extracts and compounds from *G. hypoleucum* DC against *C. violaceum* ATCC12472

Active ingredient	MIC(μg/mL)
Crude extracts	＞1000
Fraction EE	＞1000
Apigenin	500
Luteolin	500
Kaempferol	500
Quercetin	500
Rutin	＞1000

Table S3. Docking and binding energy data of each compound molecule to CviR

Group	Binding energy
C10-HLS	-3.74
Apigenin	-5.8
Luteolin	-5.46
Kaempferol	-4.82
Quercetin	-3.86
Rutin	-2.72

Table S4. Detailed data for calculations in molecular docking

gene name	center of the coordinates	size of the box
vioA	x =-1.2 y =43.1 z =4.2	x =81.5 y =58.8 z =108.1
vioB	x =-6.1 y =1.0 z =14.6	x =118.5 y =79.0 z =127.9
vioC	x =-0.4 y =-0.7 z =-4.7	x =69.0 y =58.0 z =74.1
vioD	x =16.2 y =41.9 z =32.9	x =71.2 y =62.8 z =49.5
CviR	x =-16.0 y =6.0 z =27.6	x =50.3 y =54.3 z =57.6
